# A Mobile Health Intervention to Improve Hepatitis C Outcomes Among People With Opioid Use Disorder: Protocol for a Randomized Controlled Trial

**DOI:** 10.2196/12620

**Published:** 2019-08-01

**Authors:** Karli R Hochstatter, David H Gustafson Sr, Gina Landucci, Klaren Pe-Romashko, Adam Maus, Dhavan V Shah, Quinton A Taylor, Emma K Gill, Rebecca Miller, Sarah Krechel, Ryan P Westergaard

**Affiliations:** 1 Department of Population Health Sciences School of Medicine and Public Health University of Wisconsin-Madison Madison, WI United States; 2 Department of Medicine School of Medicine and Public Health University of Wisconsin-Madison Madison, WI United States; 3 Center for Health Enhancement Systems Studies Department of Industrial and Systems Engineering University of Wiconsin-Madison Madison, WI United States; 4 AIDS Resource Center of Wisconsin Milwaukee, WI United States

**Keywords:** mHealth, eHealth, hepatitis C virus, substance use, continuum of care

## Abstract

**Background:**

People who inject drugs are at a disproportionate risk for contracting hepatitis C virus (HCV). However, use of HCV prevention and treatment services remains suboptimal among people with substance use disorders due to various health system, societal, and individual barriers. Mobile health applications offer promising strategies to support people in recovery from substance use disorders. We sought to determine whether the Addiction-Comprehensive Health Enhancement Support System (A-CHESS), an existing mobile health application for opioid use disorder, could be adapted to improve HCV screening and treatment.

**Objective:**

The goals of this paper are to describe: (1) the components and functionality of an HCV intervention incorporated into the existing A-CHESS system; and (2) how data are collected and will be used to evaluate HCV testing, linkage to care, and treatment.

**Methods:**

People with recent opioid use were enrolled in a randomized controlled trial to test whether A-CHESS reduced relapse. We developed and implemented HCV intervention content within the A-CHESS platform to simultaneously evaluate whether A-CHESS improved secondary outcomes related to HCV care. All A-CHESS users received the HCV intervention content, which includes educational information, private messages tailored to an individual’s stage of HCV care, and a public discussion forum. Data on patients’ HCV risk behaviors and stage of care were collected through quarterly telephone interviews and weekly surveys delivered through A-CHESS. The proportion of people with opioid use disorder who are HCV untested, HCV-negative, HCV antibody-positive, or HCV RNA–positive, as well as linked to care, treated and cured at baseline is described here. The 24-month follow-up is ongoing and will be completed in April 2020. Survey data will then be used to assess whether individuals who received the HCV-enhanced A-CHESS intervention were more likely to reduce risky injection behaviors, receive HCV testing, link to medical care, initiate treatment, and be cured of HCV compared to the control group.

**Results:**

Between April 2016 and April 2018, 416 individuals were enrolled and completed the baseline interview. Of these individuals, 207 were then randomly assigned to the control arm and 209 were assigned to the intervention arm. At baseline, 202 individuals (49%) self-reported ever testing HCV antibody-positive. Of those, 179 (89%) reported receiving HCV RNA confirmatory testing, 134 (66%) tested HCV RNA–positive, 125 (62%) were linked to medical care and 27 (13%) were treated and cured of HCV. Of the remaining 214 individuals who had never tested HCV antibody–positive, 129 (31%) had tested HCV antibody–negative within the past year and 85 (20%) had not been tested within the past year.

**Conclusions:**

The A-CHESS mobile health system allows for the implementation of a bundle of services as well as the collection of longitudinal data related to drug use and HCV care among people with opioid use disorders. This study will provide preliminary evidence to determine whether HCV-specific services embedded into the A-CHESS program can improve HCV outcomes for people engaged in addiction treatment.

**Trial Registration:**

ClinicalTrials.gov NCT02712034; https://clinicaltrials.gov/ct2/show/NCT02712034

**International Registered Report Identifier (IRRID):**

DERR1-10.2196/12620

## Introduction

In 2017, the United States Department of Health and Human Services declared a public health emergency to address the opioid crisis. This state of emergency followed the release of various national reports estimating that, in 2016, 2.1 million people had an opioid use disorder [1], 11.5 million people misused prescription opioids [1], 948,000 people used heroin [1], and 42,249 people died from overdosing on opioids [2], costing the United States approximately 504 billion dollars [3]. Among the many devastating consequences of the opioid epidemic is the increase in prevalence of hepatitis C virus (HCV) through the sharing of injection equipment [4,5]. The estimated prevalence of HCV antibodies among people who inject drugs in the United States is 73% [6]. According to the Centers for Disease Control and Prevention (CDC), from 2004 to 2014 acute HCV increased by 400% among people aged 18-29 and by 325% among people aged 30-39, a trend that was highly correlated with the increase in opioid addiction treatment admissions observed during the same 11-year period [7].

Despite substantial advances in HCV treatment effectiveness and tolerability, use of prevention and treatment services remains low among people with substance use disorders due to various health system, provider, societal, and individual barriers [8,9]. An estimated 72% of young people who inject drugs and are living with HCV are unaware of their HCV infection [10], with only 31% of those diagnosed ever evaluated by an HCV specialist and only a further 8% receiving antiviral treatment [11]. Few evidence-based strategies are available for improving engagement in HCV care for people with a history of injection drug use.

Stigma and discrimination, lack of access to the health care system, and complex health and social needs are among the many obstacles people with substance use disorders encounter that contribute to the observed low rates of engagement in health care compared to the general population [12,13]. Health related smartphone applications have increased in popularity for a variety of conditions [14-16], and there is a growing evidence base suggesting that they may have a role in supporting addiction treatment. They are able to both improve access to comprehensive health services as well as make recovery support, information, and monitoring available almost constantly [17,18]. The Addiction-Comprehensive Health Enhancement Support System (A-CHESS), developed at the University of Wisconsin, is a smartphone application designed to improve recovery from addiction by offering communication with peers and addiction experts, reminders and alerts to encourage therapeutic goals, individualized, addiction related, educational material, and other support services to patients. In a randomized controlled trial (RCT), A-CHESS was shown to reduce risky drinking days and enhance long term abstinence among people with alcohol use disorder, one-third of whom reported illicit drug use [18,19]. A-CHESS also reduced alcohol and opioid use in field tests with the Veterans Administration, drug courts, and among pregnant women in Appalachia [20].

Our research team previously designed and pilot tested another computerized intervention, called Hep-Net [21], to improve HCV-related outcomes among people who inject drugs and participate in harm reduction programs. The Hep-Net intervention targeted four different behavioral domains: (1) undergoing regular HCV screening; (2) using clean drug injection equipment; (3) overdose prevention; and (4) ceasing injection drug use [21]. In the context of an active RCT of A-CHESS to prevent relapse in opioid use disorder [17], our team has integrated content and functionality developed for Hep-Net into the A-CHESS platform. The goals of this substudy are to determine whether HCV-specific services embedded into the A-CHESS program can reduce risky injection behaviors and increase the frequency of HCV testing, linkage to care, and treatment for people engaged in addiction treatment. This paper describes the study protocol implemented to achieve these goals.

## Methods

### Overall Objectives

The goal of the parent RCT is to assess whether A-CHESS can prevent or delay relapse among people with opioid use disorder who are in early remission and receiving medication assisted treatment (MAT). Individuals with opioid use disorder from two addiction treatment centers in Massachusetts were randomly assigned in a 1:1 ratio to receive either MAT alone (control arm) or MAT and A-CHESS (experimental arm), stratifying on gender and site and balancing on age, level of care, and whether patients had prior substance use disorder treatment. Participants are followed for 24 months, a timeframe informed by prior research to assess the long-term impact of A-CHESS on addiction recovery with adequate power [18,22-24]. The study design, study population, recruitment, eligibility and screening process, and addiction-related services incorporated into A-CHESS have been described in the parent RCT’s published protocol [17]. We developed and implemented HCV intervention content within the A-CHESS platform to simultaneously evaluate whether A-CHESS improves secondary outcomes related to HCV care. In this manuscript, we describe the components and functionality of the HCV intervention incorporated into the existing A-CHESS system and how data are collected and will be used to evaluate HCV testing, linkage to care, and treatment.

A-CHESS contains multiple services designed to address several types of challenges facing people who need treatment and prevention services for addiction. The services are organized around the components of self-determination theory, which postulates that self-motivation and well-being are a function of three innate psychological needs: competence, autonomy, and relatedness [25]. Key A-CHESS services addressing these needs include a call for help function, cognitive behavioral therapy boosters, a GPS location tracker, tailored coping support, a counselor dashboard, coach monitored discussion groups, and HIV or HCV services [17]. A-CHESS provides a medium to disseminate educational information, opportunities to interact with peers and trained counselors, and a platform for collecting participant-level data. These existing features were utilized to collect data on patients’ HCV risk behaviors and testing history, and deliver behavior change interventions tailored to the patient’s self-reported stage of HCV care.

A variety of study team members have administrative access to the A-CHESS system (eg, coinvestigators, project managers, research assistants and addiction counselors), allowing them to view participant data and send individualized messages. A subset of the investigator team knowledgeable about HCV care (hereafter referred to as HCV research staff) were responsible for conducting the HCV substudy.

### HCV Intervention Content

#### The HCV Care Continuum Model

All individuals with A-CHESS received the HCV intervention discussed in this manuscript. The HCV intervention utilizes three A-CHESS components: information dissemination, private messaging, and a discussion forum. The content delivered through these components was developed in consideration of the so-called HCV care continuum: the process of how this disease is ideally managed from prevention, HCV antibody (Ab) screening, HCV RNA confirmation, undergoing HCV evaluation by a medical provider (ie, linkage to care), treatment initiation, retention in care, and then to achieving a sustained virologic response (ie, cure) [26]. Acknowledging that A-CHESS participants may fall anywhere along this continuum, each of the three HCV intervention components provide information and support for all stages of care, making each component relevant to the entire study population.

#### HCV Educational Information

**Figure 1:**
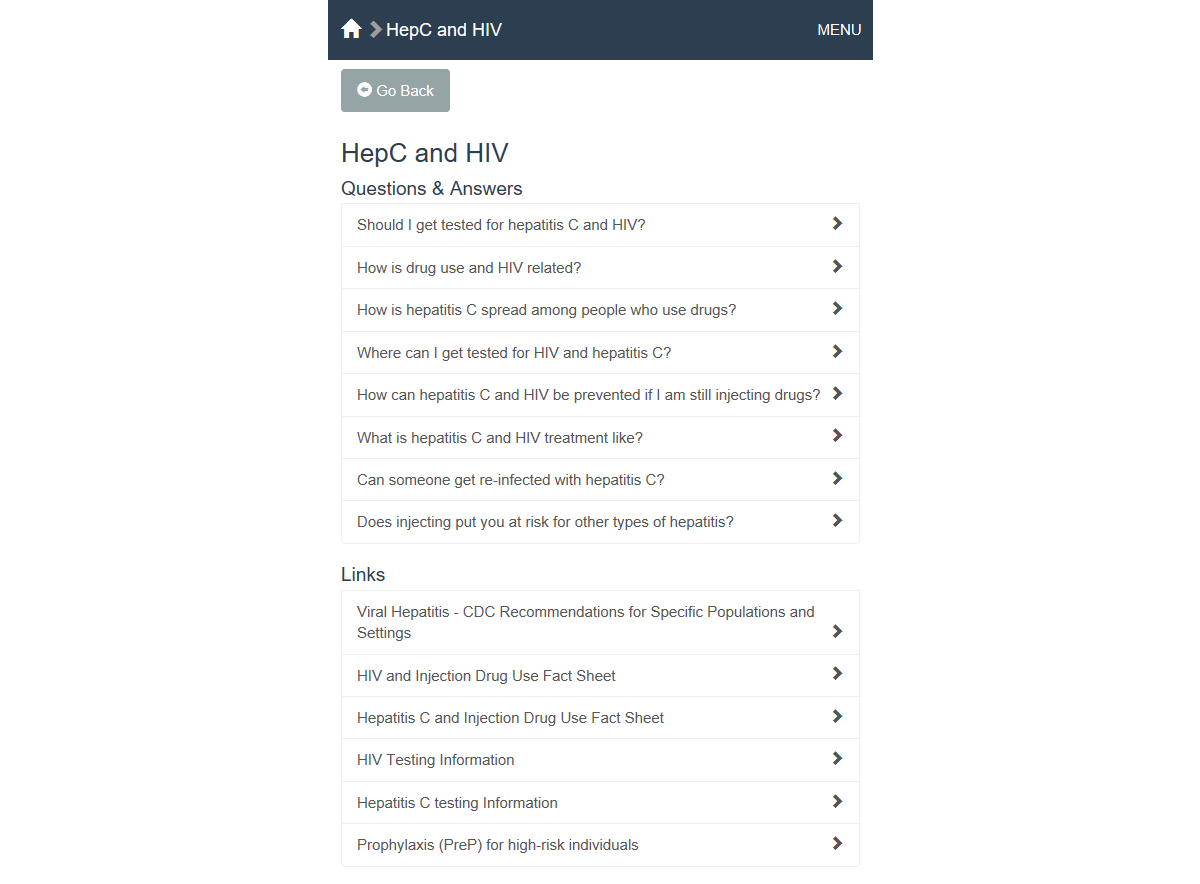
HCV and HIV educational information available on the information page of the A-CHESS app. HCV: hepatitis C virus; A-CHESS: Addiction-Comprehensive Health Enhancement Support System; CDC: Centers for Disease Control and Prevention.

Significant gaps in knowledge of HCV have been reported among people who use drugs, including a lack of knowledge regarding transmission risks, symptoms and clinical markers, as well as treatment guidelines [27-29]. Understanding that increasing knowledge of HCV is a critical component of public health interventions aimed at reducing the overall burden of HCV, educational materials were incorporated into the A-CHESS system. In addition to various topics related to opioid addiction and recovery, A-CHESS houses HCV-specific information pages that are freely navigable for users. Because injection drug use is also an important driver of the HIV epidemic, accounting for 9% of all new HIV infections [9], A-CHESS provides HIV information as well. This educational content, housed within the information tab of A-CHESS, provides answers to frequently asked questions and links to several fact sheets developed by the CDC (Figure 1). When selecting the option *Where can I get tested for HIV and Hepatitis C?*, A-CHESS users are provided with a list of screening and treatment centers near their place of residence, which is determined by the site at which they enrolled into the study. Links to CDC documents and fact sheets open a Web browser and users are warned that they are leaving the A-CHESS application. To increase awareness of this information and the frequency of views, HCV research staff often reference this content and provide direct links through posts on public discussion boards and private messaging conversations.

News articles related to the intersecting epidemics of opioid injections and infectious diseases are also posted on the information tab (eg, HCV outbreaks among people who inject drugs, the release of new pharmaceutical treatments, important policy changes, and so on). Videos from people affected by these epidemics and expert medical providers working in the field are also on A-CHESS and serve as a powerful means to disseminate information to A-CHESS users.

#### Private Messaging

Mobile health messaging interventions, such as texting, present promising strategies to increase engagement in health care. These interventions may serve a variety of purposes, including reminders, alerts, education, motivation, and prevention. A large body of literature exists that demonstrates the effectiveness of text message interventions on health outcomes, including the intent to obtain and complete the HPV vaccination series [30-32], uptake of HIV testing [33-35], improved knowledge of sexually transmitted infections (STIs) [36], and increased attendance at HIV appointments [33,37,38]. Although receiving information on HCV and other STIs through text messages has generally been well-accepted by patients [39-41], the effectiveness of mobile health messaging interventions on HCV care engagement have not been well studied. A-CHESS provides a private messaging service that functions very similarly to the standard texting application provided by smartphones, in which users may share content that is visible only to one or more intended recipients. Participants may opt in to receive automated (push) notifications on their smartphones when a private message is received in A-CHESS, when another user posts in a public discussion forum, or both.

HCV research staff use the private messaging feature to send messages related to goals associated with the HCV care continuum. To create a systematic approach for these private conversations, a manual was developed so that individuals in the same HCV stage of care receive the same initial message. Subsequent messages are then guided by each individual’s unique response, but towards the same goal of helping each individual advance appropriately along the HCV care continuum. Participants received a minimum of 3 private messages from HCV research staff. When participants responded using the private messaging platform, the HCV research team received an automated e-mail warning them of the new message. This prompted staff to log into A-CHESS and respond at their earliest convince. When staff did not receive a response from the participant, a standardized follow-up message was sent approximately 1 month later.

Because eligibility criteria did not specify a method of opioid use, there are many participants who have not engaged in any injection risk behaviors. If an individual shares that they have never injected drugs and does not believe they are at risk of HCV, research staff express an understanding of their low risk, share information on other means of transmission (eg, sexual contact, tattoos and piercing, and so on), encourage continued preventative actions, recommend one-time testing, and direct them to the educational content to ensure they have information to protect themselves from contracting HCV. Research staff also reference the baseline survey, discussed below, to assess each participant’s date of birth. If the participant’s date of birth is between 1945 and 1965, the CDC recommendation for all baby boomers to receive a one-time HCV test is shared [42]. Individuals who report being diagnosed with HCV can pose questions to HCV research staff who are familiar with treatment resources in their community. A coinvestigator (an infectious disease physician) maintains a user account, through which they can share health information of general interest to the group and respond to participants’ nonurgent health questions related to HCV testing and treatment options.

#### Discussion Boards

Peer-based education has been shown to be an effective method for reducing risky injection behaviors and preventing HIV transmission [43-45], as well as enhancing HCV treatment knowledge and initiation [46,47]. A-CHESS contains several discussion boards to foster communication of different educational and emotional content. Discussion boards allow users to create and view messages that are accessible to all or only to a subset of study participants. A public group provides a forum for participants to share experiences and engage in conversations on general topics. Separate, treatment specific discussion groups allow participants to interact with other individuals receiving similar forms of MAT, including methadone, buprenorphine, or injectable naltrexone. In these groups, participants are encouraged to discuss experiences that may be specific to their form of MAT, such as side effects, dosage, and adherence challenges. A third public discussion board, named Staying Healthy, is dedicated to discussions surrounding HCV. A-CHESS users use this board to ask infectious disease related questions, share HCV treatment experiences, and discuss barriers to both testing and treatment. HCV research staff also engage in these conversations to remind A-CHESS users of the importance of being tested for HCV, encourage healthy behaviors, and stimulate discussion related to such topics. Upon study enrollment, A-CHESS users create a username of their choice, allowing them to choose an anonymous name that is unidentifiable if that makes them more comfortable when participating in these public discussions. All public discussion threads are monitored regularly by two project managers for appropriate use.

### Functionality for Providers

The Moderator Dashboard provides a snapshot to study personnel of key metrics for each study participant, including their treatment center, date of enrollment, HCV status, when they last logged onto the app, A-CHESS use for the past 30 days, the past 25 private messages exchanged between the participant and study personnel, and whether or not the participant viewed the study personnel’s most recently delivered private message. Private messages may also be sent directly from the moderator dashboard. Having this information readily available on one screen allows HCV research staff to quickly deliver private messages while accounting for contextual characteristics that are not displayed on the private messaging platform. Staff can use this dashboard to sort and view participants according to who they most or least recently privately messaged, who was most or least recently active on the app, and other individual characteristics. Participants can also be sorted according to their stage in the HCV care continuum in order to facilitate sending private messages in a systematic way. For example, an HCV research staff member could choose to only view study participants classified as HCV-untested, and then send a private message to each individual encouraging testing.

### Data Collection Tools

#### Baseline Study Assessment and Quarterly Telephone Interviews

**Figure 2:**
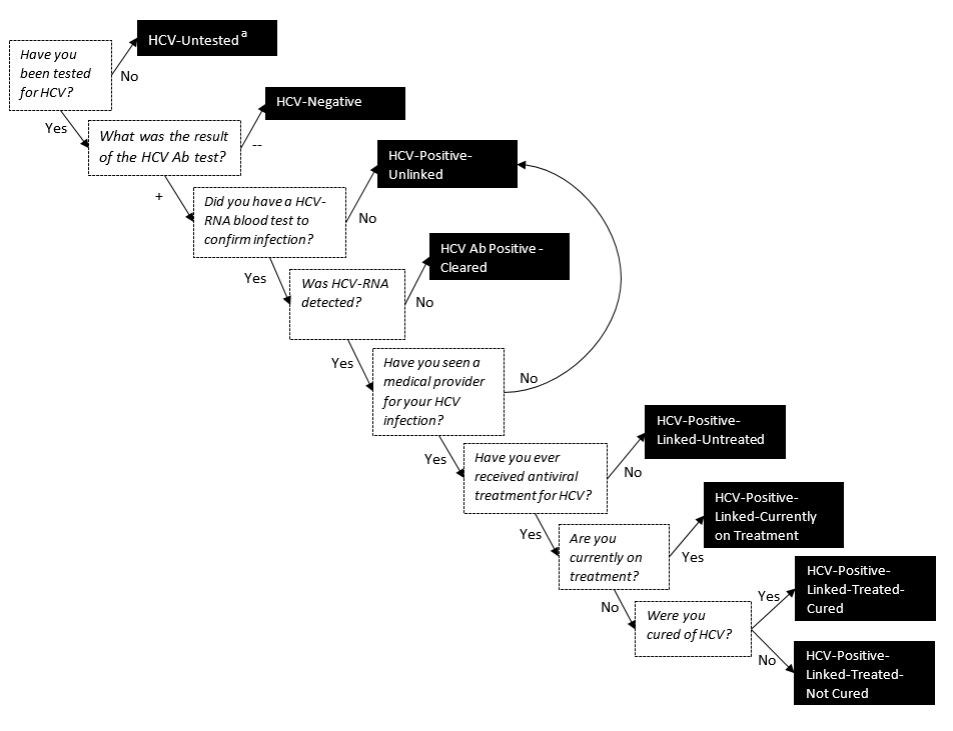
Questions (in white boxes) asked during the baseline interview that inform the assignment of an HCV stage of care (in black boxes). "a" indicates that individuals were also considered HCV-Untested if they had not been tested in the past year, if they did not know whether they have ever been tested, or if they had reported injecting drugs since their last HCV test. HCV: hepatitis C virus; Ab: antibody.

Baseline questionnaires with both intervention and control participants were conducted over the phone by two study coordinators at the University of Wisconsin-Madison. Information collected at baseline included demographic items, opiate use history, chronic pain, comorbid conditions, and HCV status. Participants were asked at baseline whether they had ever been tested for HCV, as well as the date and result of their last test. If the results from this test were positive, linkage to care and HCV treatment initiation and completion were assessed. Participants were then assigned a baseline stage of HCV care using the logic outlined in Figure 2. Time since last HCV test, whether they had injected drugs since their last test, and if they did not know the answer to questions was taken into consideration when assigning stages of HCV care (see Multimedia Appendix 1). Individuals were also considered HCV-untested if they had not been tested in the past year, if they did not know whether they have ever been tested, or if they had reported injecting drugs since their last HCV test. Both intervention and control participants also received calls from the same two study coordinators quarterly, at months 4, 8, 12, 16, 20 and 24. Follow-up interview questions assessed the same domains as the baseline questionnaire, allowing for the recognition of changes over time.

#### Weekly Check-In Surveys

In addition to the baseline and quarterly telephone interviews, intervention participants completed short electronic surveys delivered weekly through the A-CHESS app. The brief addiction monitor (BAM) [48], adapted for the smartphone interface using visual analog scales, provided the foundation for weekly assessments of general health status, mood, social support, MAT adherence, and progress with addiction recovery. In addition to these determinants of health, the weekly check-in captured HCV information which could be used to measure progression through the entire sequence of the HCV care continuum and the speed at which individuals received appropriate follow-up testing, got linked to medical care, initiated treatment, and were cured. Logic incorporated into the server back-end of the A-CHESS system was designed to deliver HCV-related questions for the first weekly survey according to the baseline stage of care the participants were assigned during the initial telephone interview. It then updated the HCV stage based on answers to the weekly survey (see Multimedia Appendix 2) and delivered subsequent weekly survey questions according to the newly assigned stage of HCV care. This logic ensured that individuals who previously tested negative for HCV and who continued to engage in risky injection behaviors were asked to repeat testing on a regular basis.

### Data Analysis

We used data provided during the baseline telephone interviews to characterize the HCV continuum of care for all participants at the time of enrollment.

The effectiveness of this HCV intervention within the A-CHESS framework will be assessed upon completion of the 24-month follow-up. Four primary outcomes will be examined: undergoing HCV testing, linking to HCV medical care, initiating HCV treatment, and achieving a sustained virologic response. The quarterly telephone interviews, conducted by both intervention and control arms, allows for accurate comparisons between groups. We will conduct four separate binomial logistic regression analyses to assess whether individuals who received A-CHESS were more likely to achieve each of the primary outcomes by 24 months compared to those who did not receive A-CHESS, using an intention-to-treat approach and adjusting for the baseline stage of HCV care. The weekly survey information will allow for a more time-sensitive estimate of the rates at which individuals who use A-CHESS advance along the HCV care continuum.

Upon study completion, the frequency that different HCV components were utilized will also be analyzed to assess how each component is related to improved HCV outcomes among A-CHESS participants. This will allow us to understand the effectiveness of the educational information section, private messaging platform, and public discussion forums individually, as well as estimate the time elapsed between accessing different A-CHESS services and achieving HCV outcomes. Furthermore, although the weekly surveys and quarterly telephone interviews serve as data collection tools, there may be an intervention effect by reminding people to get tested, see a medical provider, and initiate and adhere to treatment. Whether these data collection tools had an intervention effect will be measured by comparing those who did and did not complete the surveys. Understanding the effectiveness of intervention components will inform future, more efficient A-CHESS models.

## Results

Participant recruitment occurred during the first 2 years of the study, between April 2016 and April 2018. During this period, 416 individuals were enrolled and completed the baseline survey. Then, 207 were randomly assigned to the control group and 209 were assigned to receive A-CHESS. The 24-month follow-up of enrolled participants is expected to continue until April 2020.

At baseline, 202 individuals (49%) reported ever testing HCV Ab-positive. Of those, 179 (89%) reported receiving HCV RNA confirmatory testing, of which 134 (75%) tested HCV RNA–positive. Among those who reported testing HCV RNA–positive, 125 (93%) reported seeing a medical provider and 27 (20%) had received HCV treatment and achieved a sustained virologic response (Table 1). Of the remaining 214 individuals who had never tested HCV Ab-positive, 129 (60%) individuals reported testing HCV Ab-negative within the past year and 85 (40%) reported not being tested within the past year.

Four (1%) individuals were HIV-positive at the time of study enrollment. All four individuals were on antiretroviral medications and reported strong adherence.

**Table 1:** Hepatitis C virus continuum of care at baseline (N=416).

HCV^a^ status			n (%)
**Tested HCV Ab^b^–positive**			202 (49)
	**Tested HCV RNA–positive**		134 (75)
		Saw a medical provider	125 (93)
		Received HCV treatment	27 (20)
**Not tested HCV Ab–positive**			214 (51)
	HCV Ab–negative		129 (60)
	Not tested		85 (40)

^a^HCV: hepatitis C virus

^b^Ab: antibody

## Discussion

### Overview

The overall goal of this novel mobile health system is to support individuals recovering from opioid addiction and improve screening, linkage to care, and treatment rates for HCV, the most common infectious disease burdening people who have a history of opioid use disorder [49]. Baseline data collected from the complete study cohort reveals that nearly half of all study participants (49%) have tested HCV Ab-positive, while few are receiving treatment. An additional 20% of the study population had not been screened for HCV in the past year. These results demonstrate the strong need for HCV screening and treatment interventions among people with opioid use disorders and assures us that this intervention is being implemented among a population with significant potential to benefit. The overall impact that this innovative mobile health system has on HCV-related outcomes will be assessed after 24 months of follow-up interviews and weekly check-in surveys have been collected.

A prior study that examined provider and staff perceptions of A-CHESS implementation identified several facilitating factors of implementation that also served as important strategies for the implementation of the HCV intervention. Among these factors was the creation of a dedicated internal HCV team with clear roles and responsibilities to lead implementation, the orientation of clients to the content early to build awareness and interest, and the building of a separate discussion forum to stimulate conversation on HCV and build client engagement. These authors also highlighted the importance of collaborating with the mobile app development team to address technical issues [50]. Our close collaboration and weekly meetings with the app development team was crucial for rapidly responding to early implementation issues related to the survey logic and manually changing a participant’s assigned stage of HCV care based on private messaging conversations.

### Limitations

The generalizability of this study may be limited because participant recruitment was restricted to two addiction treatment centers in the state of Massachusetts. In Massachusetts, fee-for-service and managed care organizations do not have any liver damage, sobriety, or prescriber restrictions [51]. The effect of A-CHESS on HCV outcomes in states with more restricted treatment access may be limited and should be studied independently. Generalizability is also limited to individuals actively and newly engaged in treatment for opioid use disorder. Upon study completion, changes along the HCV care continuum can be compared across subgroups of participants receiving methadone, injectable naltrexone, and buprenorphine, but cannot be compared to individuals receiving no or other treatment regimens, those suffering from other forms of substance abuse, or those at other stages in addiction recovery.

Another limitation of this study is the use of self-reported stages of HCV care. Confusion regarding modes of transmission, the natural history of HCV infection, and interpretation of different HCV tests is common [27-29,52]. In an effort to capture these uncertainties, survey responses allowed individuals to select *I don’t know* as an answer choice to HCV questions (See the Multimedia Appendix 1).

At the time of the baseline assessment, 93% of HCV RNA–positive individuals reported they had seen a medical provider for HCV, a level substantially higher than what had been reported in prior studies [26,53,54]. Because the survey questions did not specifically ask whether participants saw a provider specifically to discuss starting HCV treatment, our study may overestimate true linkage to HCV care. Future studies should specify details of the clinical encounter that are of interest. Fortunately, the quarterly follow-up surveys do ask if individuals have received any tests to determine whether they have evidence of liver disease, and these responses will allow us to estimate whether or not individuals received some clinical evaluation to assess their candidacy for HCV treatment after enrollment.

Several intrapersonal characteristics of an individual that are not measured through A-CHESS may influence engagement in care. For example, self-control, organization, and self-awareness are all facets of conscientiousness believed to influence engagement in healthcare [55]. Additionally, responses to mobile health interventions are not expected to be uniform across the study population as individuals differ significantly in their ability and willingness to engage in online communication, a construct that is difficult to measure.

### Conclusion

The A-CHESS mobile health system allows for the implementation of a bundle of services and the collection of longitudinal data related to drug use and HCV care among people with opioid use disorders. This study will contribute novel data to better understand whether mobile health applications can support the complex health needs of people affected by the intersecting epidemics of opioid addiction and HCV infection. If effective, this mobile health application has the potential to improve HCV-related outcomes among hard to reach populations that are often disengaged from health care.

## Appendix

Multimedia Appendix 1 How to assign participants to initial HCV stage based on baseline questionnaire.

Multimedia Appendix 2 How update HCV stages based on weekly check-in questions.

Multimedia Appendix 3 Peer-reviewer report from the National Institute on Drug Abuse.

## Abbreviations

Ab: antibody
A-CHESS: Addiction-Comprehensive Health Enhancement Support System
BAM: brief addiction monitor
CDC: Centers for Disease Control and Prevention
HCV: hepatitis C virus
MAT: medication assisted treatment
RCT: randomized controlled trial
STI: sexually transmitted infection
