# Supplementary material for: A Mobile Health Intervention to Improve Hepatitis C Outcomes Among People With Opioid Use Disorder: Protocol for a Randomized Controlled Trial
Source: JMIR Res Protoc. 2019 Aug 1;8(8):e12620. doi: 10.2196/12620 (PMC6694728; doi:10.2196/12620)
Supplement: Multimedia Appendix 1 [file resprot_v8i8e12620_app1.docx]

**How to assign participants to initial HCV stage based on baseline questionnaire:**

________________________________________________________________________________________

[HCV Untested]:

[Ever tested for HCV] = No or don’t know

OR

[Last HCV test date] = greater than 1 year ago

AND [Last HCV test result] = "Negative" or "don't know"

OR

[Last HCV test date] = less than 1 year ago

AND [Last HCV test result] =  "don't know"

OR

[Last HCV test date] = greater than 1 year ago

AND [Last HCV test result = "Positive"

AND [Blood test to confirm] = "Yes, clear/not infected"

OR

[Inject drugs in past month] = Yes or DK

AND [Last HCV test result] = "Negative" or "don't know"

OR

[Inject drugs in past month] = Yes

AND [Last HCV test result = "Positive"

AND [Blood test to confirm] = "Yes, clear/not infected"

________________________________________________________________________________________

[HCV Negative]:

[Last HCV test result] = negative

AND

[Inject drugs in past month] = No

________________________________________________________________________________________

[HCVPos/unlinked]:

If: [Last HCV test result] = positive

AND

[Saw HCV provider] = No or DK

OR if: [Last HCV test result] = positive

AND

[Blood test to confirm]= No or DK

OR IF: [Ever on HCV meds] = Yes

AND

[Cured or Cleared] = No or DK

AND

[Currently on HCV meds] = No

________________________________________________________________________________________

[HCVPos/linked/untreated]:

If: [Last HCV test result] = positive

AND

[Saw HCV provider] = yes

AND

[Blood test to confirm] = yes - still infected

AND

[Ever on HCV meds] = No or DK

________________________________________________________________________________________

[HCVPos/linked/on treatment]

If: [Currently on HCV meds] = yes

________________________________________________________________________________________

[HCVPos/linked/treated/not cured]

If: [Last HCV test result] = positive

AND

[Saw HCV provider] = yes

AND

[Ever on HCV meds] = yes

AND

[Currently on HCV meds] = no

AND

[Cured/cleared] = no

________________________________________________________________________________________

[HCVPos/linked/treated/cured]

If: [Last HCV test result] = positive

AND

[Saw HCV provider] = yes

AND

[Ever on HCV meds] = yes

AND

[Cured] = yes

________________________________________________________________________________________________________________________________________________________________________________

[HCVPos/linked/cleared]

If: [Last HCV test result] = positive

AND

[Blood test to confirm] = Yes – Virus NOT still in blood
