# Supplementary material for: A Mobile Health Intervention to Improve Hepatitis C Outcomes Among People With Opioid Use Disorder: Protocol for a Randomized Controlled Trial
Source: JMIR Res Protoc. 2019 Aug 1;8(8):e12620. doi: 10.2196/12620 (PMC6694728; doi:10.2196/12620)
Supplement: Multimedia Appendix 2 [file resprot_v8i8e12620_app2.docx]

**How update HCV stages based on weekly check-in questions:**

________________________________________________________________________________________

[HCV Untested]:

1. [HCV Negative]: update to [HCV Untested] if [Last risky injecting date] is later than [last HCV test date].
2. [HCVPos/linked/treated]: update to [HCV Untested] if [Last risky injecting date] is later than [last HCV test date].
3. [HCVPos/linked/cleared]: update to [HCV Untested] if [Last risky injecting date] is later than [last HCV test date].

*In other words, participants at these 3 stages will revert to untested if they start injecting/sharing again.*

________________________________________________________________________________________

[HCV Negative]:

If current stage = [HCV Untested], update to [HCV Negative] when new response to “Have you been tested . . .” is Yes AND [Last HCV test result] = negative/nonreactive

_______________________________________________________________________________________

[HCVPos/unlinked]:

1. If current stage = [HCV Untested], update to [HCVPos/unlinked] if:

- Response to “Have you been tested . . .” is Yes AND [Last HCV test result] = Positive

AND

- Response to “Have you seen a medical provider. . .” is No

1. If current stage = [HCVPos/linked], update to [HCVPos/unlinked] if:

Response to “Have you been tested . . .” is Yes AND [Last HCV test result] = Positive

AND

Response to “Have you seen a medical provider. . .” is Yes

AND

Response to “Have you had a blood test . . . “ is No

_______________________________________________________________________________________

[HCVPos/linked/untreated]:

1. If current stage = [HCV Untested], update to [HCVPos/linked/untreated] if:

- Response to “Have you been tested . . .” is Yes AND [Last HCV test result] = Positive

AND

- Response to “Have you seen a medical provider. . .” is Yes

AND

- Response to “Have you had a blood test . . . “ is Yes – Virus is still in my blood

AND

- Response to “Are you currently taking. . . “ is No

1. If current stage = [HCVPos/unlinked], update to [HCVPos/linked/untreated] if:

- Response to “Have you seen a medical provider. . .” is Yes

AND

- Response to “Have you had a blood test . . . “ is Yes – Virus is still in my blood

AND

- Response to “Are you currently taking. . . “ is No

________________________________________________________________________________________

[HCVPos/linked/on treatment]

1. If current stage = [HCVPos/unlinked], update to [HCVPos/linked/on treatment] if:

- Response to “Have you seen a medical provider. . .” is Yes

AND

- Response to “Have you had a blood test . . . “ is Yes – Virus is still in my blood

AND

- Response to “Are you currently taking . . .” is Yes

1. If current stage = [HCVPos/linked/untreated], update to [HCVPos/linked/on treatment] if:

- Response to “Are you currently taking . . .” is Yes

________________________________________________________________________________________

[HCVPos/linked/treated/not cured]

1. If current stage = [HCVPos/linked/on treatment] update to [HCVPos/linked/treated] if:

- Response to “Are you currently taking . . .” is No

AND

- Response to “Were you cured . . . “ is No

________________________________________________________________________________________

[HCVPos/linked/treated/cured]

1. If current stage = [HCVPos/linked/on treatment] update to [HCVPos/linked/treated] if:

- Response to “Are you currently taking . . .” is No

AND

- Response to “Were you cured . . . “ is Yes
