# Supplementary material for: A Mobile Health Intervention to Improve Hepatitis C Outcomes Among People With Opioid Use Disorder: Protocol for a Randomized Controlled Trial
Source: JMIR Res Protoc. 2019 Aug 1;8(8):e12620. doi: 10.2196/12620 (PMC6694728; doi:10.2196/12620)
Supplement: Multimedia Appendix 3 [file resprot_v8i8e12620_app3.pdf]

Response To Concerns From Reviewers Of
1 R01 DA040449-01; David H. Gustafson Phd And Kimberly Johnson PhD.

We were excited that the study section concluded that that "the proposed research is excellent, highly significant and innovative and is likely to have a high potential impact in improving long-term treatment outcomes for opioid use disorders, with the potential for broad scale translation." Still the reviewers provided important points to consider.

It is important to note that none of the Responses to those concerns entail any significant change in the analytic strategies or goals as originally proposed. We are simply providing more information to clarify certain aspects of our approach.

Concern: Aims were separated that could have been listed together. The Resume and Summary of Discussion indicates that the panel eventually decided that the number of aims was not a problem. Some on the panel might have preferred that aims be structured as a function of the analytic question being addressed. If one were to organize the aims around analytic questions, the Aims could be restructured as:

- **Aim 1** (Primary). Detect the difference in illicit opioid use between patients who have MAT + A-CHESS vs. MAT alone.
- **Aim 2** (Secondary). Detect differences between patients who have MAT + A-CHESS vs. MAT alone in terms of: quality of life; retention in treatment; unscheduled use of health services; and, for HCV and HIV screening rates, risk behaviors, testing, and among those infected, treatment initiation.
- **Aim 3** (Secondary) Identify mediators of the effects of MAT + A-CHESS vs. MAT on illicit opioid use; key candidate mediators will include the 3 constructs of self-determination theory (competence, relatedness, and intrinsic motivation), communication style, use of A-CHESS, and negative affect.
- **Aim 4** (Secondary). Determine person factors that moderate the impact of MAT + A-CHESS vs. MAT alone (e.g., gender, SUD severity).

In this representation, the number of aims has been reduced by 1/3rd. Note that all of the secondary aims differ from one another in *both* substantive concern as well as analytic method. Also, while we do plan to characterize the struggles we encounter in implementing and sustaining the interventions, we no longer list this as a formal aim.

Concern. The investigative team lacks an experienced qualitative methodologist. Dr. Nora Jacobson, Senior Scientist and Qualitative Methodologist at our Institute for Clinical and Translational Research will mentor Dr. Alagoz and guide the qualitative study design, data collection, and data analysis process. Attached you can find her biosketch. Dr. Jacobson is an interpretive social scientist with more than 15 years of health service research experience who uses qualitative and community-based participatory methods to study the delivery of health services and the development of health policy. Over the last decade much of her research focused on how healthcare organizations implement innovative practices. In addition, she has been involved in several studies that examined contextual factors that promote knowledge transfer and exchange between applied health services, researchers and health system decision-makers. As the qualitative methodologist for the Institute for Clinical and Translational Research (ICTR) at the University of Wisconsin-Madison, she provides consultative research support to ICTR investigators and we believe is well qualified to mentor Dr. Alagoz. It should be noted that Dr. Alagoz has been promoted to the position of Assistant Scientist in our Center.

Concern. The aims were "underdeveloped methodologically". We were somewhat unclear about this concern. However, we interpreted that characterization to mean that the panel wished for greater specificity regarding particular aspects of the proposed analyses. In particular, we believe that the reviewers were interested in more details about the nature of the dependent variable to be analyzed for the Primary Aim, about the qualitative assessment and analyses, and the nature of the mediational analyses.

1. **Dependent variable.** With regard to the nature of the dependent variable in the Primary Aim analysis, we state on p. 7 of the application that this analysis will "Detect the difference in illicit opioid use between patients who have MAT + A-CHESS vs. MAT alone (primary aim). *Hypothesis:* Patients with MAT + A-CHESS will use fewer illicit opioids." However, we can understand that the panel may have wanted clarification of how we would measure the dependent variable. Self-reported drug use days will be analyzed in 30-day periods. For Baseline, the TimeLine FollowBack[1] (TLFB) for the last 30 days prior to

admission will be obtained and a urine drug screen (CTN-approved drug use outcome measures)[1]. For follow-up assessments the TLFB for the previous120 days will be obtained, corroborated by a urine screen. The TLFB has been successfully used to obtain drug use data for extended periods of time and with poly drug using patients[2].

2. **Mediational analyses**, we believe that review panel might have wanted more information about the timing of the mediator assessments relative to the outcome assessments (will there be overlap in the assessment of the mediators and outcome?) and whether the mediation analytic plan takes into account the fact that the various mediators might overlap with one another in their relations with treatment and outcome. To clarify, our mediational models will involve no temporal overlap in the collection of mediators and the outcome. The mediational variables will all be collected in the first 2 visits (at the 4 & 8 month visits) while the outcome (illicit drug use) will collected at the 12, 16, 20, & 24 month visits. Moreover, because mediator-outcome relations might reflect the effects of drug use while the mediator is being assessed (e.g., drug use might suppress ratings of competence), drug use that occurs during the mediator assessment period will be covaried out of models to examine and control its influence. Moreover, in order to assess the nonorthogonality of the mediators, (which seems quite likely with the self-determination variables), we will use multiple mediator analyses based on a Bayesian approach illustrated in Yuan and MacKinnon (2009)[3]. This Bayesian estimation of the mediational models can be implemented through Markov Chain Monte Carlo (MCMC) techniques. Unlike more traditional estimation methods such as maximum likelihood or least squares methods, for example, MCMC methods rely on sampling techniques to estimate model parameters and resulting mediation effects (i.e., iterative sampling from the parameter distributions is used to estimate CIs to identify significant effects). An appealing feature of the methodology is its relative ease of implementation, particularly for complex statistical models. Similar to Yuan and MacKinnon, we will implement MCMC using WinBUGS 1.4[4]. The multiple mediator models will be conducted with only those mediators shown to be significant in univariate models. See Bolt et al., (2012) [5] for our previous application of this analytic approach.
3. **Qualitative analysis.** The Qualitative Content Analysis (QCA)[6][7] (Krippendorf, 2013; Schreier, 2012 that we propose to employ for our data analysis follows a consistent set of steps for each set of data (interviews and case study data) and with each coder. These steps and our implementation of them include: a) Deciding on the research questions (the questions are stated in the proposal), b) Selecting the data collection tools (either interview data or case study data based on which research questions we would like to address), c) Building a coding scheme (our coding scheme will be data driven using a method built on grounded theory; hence we will not use an a-priori scheme), d) Dividing the data into units of coding (in order to refine our unit of analysis, we will use *sentences* as our unit of analysis. *Sentences* will be useful in calculating inter-rater reliability and in keeping our coding consistent among coders.), e) Testing the coding scheme (we will pilot test and modify our coding scheme on a subset of data so that our codes are detailed enough to capture our research questions), f) Main analysis (coders will code the data independently using the modified coding scheme.) and g) Interpreting and presenting findings.

Concern. Data collection burden on patients was too high. The reviewers are correct. The burden from each follow-up interview was 285 items. We reduced the burden by 50% (to 144 items) by eliminating multiple scales for one concept and using revised (reduced) scales instead of original scales. All scales are still validated. Specifically, our changes include:

---

[1] Donovan D, Bigelow G, Brigham G, et al. (2012). Primary outcome indices in illicit drug dependence treatment research: systematic approach to selection and measurement of drug use end-points in clinical trials. Addiction. 107(4): 694-708.

[2] Sobell LC, Brown J, Leo GI, Sobell MB. The reliability of the Alcohol Timeline Followback when administered by telephone and by computer. Drug and Alcohol Dependence. 1996;42:49–54.

[3] Yuan, Y., & MacKinnon, D. P. (2009). Bayesian mediation analysis. *Psychological Methods, 14,* 301–322. doi:10.1037/a0016972

[4] Spiegelhalter, D. J. (2008). Understanding uncertainty. *Annals of Family Medicine, 6,* 196–197. doi:10.1370/afm.848

[5] Bolt DM, Piper ME, Theobald WE, Baker TB. Why two smoking cessation agents work better than one: Role of craving suppression. *J Consult Clin Psychol.* 2012;80(1):54-65. PMCID: PMC3265654

[6] Krippendorff, K., Content analysis: An introduction to its methodology. 2012: Sage.

[7] Schreier, M., Qualitative Content Analysis in Practice. 2012, Thousand Oaks, CA: Sage.

- Using the SF-12 quality of life scale rather than the 26 items WHOQO-BREF scale;
- Using the Revised Dyadic Adjustment Scale (14 items) instead of the original (24 items);
- Using the Adjective Rating Scale of Withdrawal (16 items) but not the 16 item Distress Tolerance Scale.
- Measuring each of the four mediators with one instrument rather than two , with
  - Relatedness measured by our 6-item bonding scale (drop the Important People and Activities scale – 19 items);
  - Intrinsic motivation, measured by the intrinsic motivation subscale (4 items) of the Client Motivation for Therapy scale and drop the Treatment Self Regulation Questionnaire (15 items) and the CBI (35 items)
  - Competence, measured by the revised 8-item Drug Taking Confidence Scale rather than the 50 item original.

Concern : Dropout rates may be underestimated.. In our RCT, 88 of the patients were using opioids as well as alcohol; 261 were not using opioids. We compared the post-test interview response rate of opioid using patients to the patients that did not use opioids. The non-opioid-using the patients' response rates were: 94.3% at 4 months; 90.6% at 8 months and 86.7% at 12 months. The opioid using patients' response rates were 91.2%, 86%, and 79.1%. The response rates declined in a relatively linear fashion in both groups, with reductions of about 5% in each period. We assumed a 65% response rate at 24 months by continuing the drop off at a 5% rate for each of the 3 succeeding periods from 79% to 74% to 69% to 64%. Hence we believe it is likely that by the end of the study we will still be able to reach 65% of patients originally enrolled.

Concern: The incorporation of services related to HIV and hepatitis C was a "distracting" addition to the proposal, We believe there are two ways the HIV/HCV component adds value to the intervention. 1) The prevalence of HIV/HCV infection is high among opioid using populations, yet most addiction treatment centers do not perform any routine testing. Facilitating protocol-driven approaches to infectious disease screening and linkage to care is a promising function of mHealth interventions. In the present study, bundling HIV/HCV services with A-CHESS could help the addiction treatment centers fulfill an obligation to screen a high risk population for two serious but highly-treatable conditions. 2) Screening for HIV-HCV is consistent with the project's overall goal of improving access to comprehensive health services for opioid-dependent patients, rather than focusing narrowly on promoting abstinence from opioids. We recognize that despite availability of evidence-based interventions, many patients who have injected opioids will relapse. The bundled intervention seeks to meet a public health goal of reducing the number of people who are infected with HIV or HCV but are unaware, and therefore continue to place others at risk.

Concern. The HIV/HCV component was not completely weaved into the entire application. We acknowledge that as the newest components of the A-CHESS system, the services related to HIV/HCV testing and linkage to care are the least well-integrated into the existing application, and therefore additional programming resources will be required in year 1 of the study. We have, however, accounted for this need in our work plan and associated budget. Since the proposal was submitted, Dr. Westergaard's team has continued to accumulate experience with mHealth approaches to HIV/HCV screening and risk reduction through other NIH-funded research projects. This project will continue to strengthen the collaboration among the CHESS team, Dr. Westergaard and the UW Division of Infectious Diseases. We believe that the way that we have reframed the aims has allowed us to better demonstrate how the HIV/HCV outcomes fit into our study design.

Concern. The cost analysis was underdeveloped regarding the measurement and analysis of healthcare utilization. We appreciate the opportunity to provide further details on our plan. Our proposed analysis of health services utilization was motivated by the potential A-CHESS has shown to reduce the kinds of costly, unscheduled health services utilization associated with relapse; in a field test with U.S. military veterans, A-CHESS users decreased re-hospitalizations due to relapse by 71%. Our approach to measuring and analyzing health utilization data is adapted from McCollister & French's 2003 analysis of the economic benefit of addiction interventions[8]. It defined several categories for healthcare utilization, including: Therapeutic Community Treatment (day), Emergency room (visit), Hospital detox (day), Short term residential treatment (day), Non-residential treatment (visit), Outpatient treatment (visit), Individual psychotherapy (visit), Methadone maintenance treatment (day), Outpatient psychological treatment (visit), and In-patient

---

[8] McCollister, K. E., & French, M. T. (2003). The relative contribution of outcome domains in the total economic benefit of addiction interventions: a review of first findings. *Addiction*, *98*(12), 1647-1659.

psychological treatment (day). Of these categories, we will include the following as unscheduled use: Emergency room (visit), Hospital detox (day), and Short term residential treatment (day). To these categories, we will also add a category for urgent care visits and apply cost estimates derived from a national survey of urgent care clinics (Weinick et al., 2009)[9]. We will include use of hospitals, emergency rooms, and urgent care for any reason (i.e., possibly but not necessarily related to substance use). It is worth noting that many of the sources for cost estimates used in McCollister & French's analysis are dated (some going back as far as 1996); we will derive up-to-date cost estimates for ER visits, hospitalizations, and residential treatment using data from the American Hospital Association and American Medical Association. Using patient surveys, we will also assess patients' use of outpatient addiction treatment services following relapse, using the categories of outpatient addiction care outlined by McCollister and French (above). Costs for outpatient addiction treatment associated with relapse will be estimated using service cost estimates provided in French et al.'s 2008 national survey of 110 substance abuse treatment programs[10] adjusted for inflation.

---

[9] Weinick, R. M., Bristol, S. J., & DesRoches, C. M. (2009). Urgent care centers in the US: Findings from a national survey. *BMC health services research*, *9*(1), 79

[10] French, M. T., Popovici, I., & Tapsell, L. (2008). The economic costs of substance abuse treatment: Updated estimates and cost bands for program assessment and reimbursement. *Journal of substance abuse treatment*, *35*(4), 462-469.
